# SHIELD: An AI Framework for Skeletal Health Intelligence and Early Lesion Detection to Improve Orthopedic Referrals

**DOI:** 10.1007/s10916-026-02373-6

**Published:** 2026-04-13

**Authors:** Abbas Alili, Ava M. McKane, Muhammet F. Demir, Cynthia L. Emory, Metin N. Gurcan

**Affiliations:** 1 Center for Artificial Intelligence Research, Wake Forest University School of Medicine, Winston-Salem, NC USA; 2 Department of Orthopaedic Surgery and Rehabilitation, Wake Forest University School of Medicine, Winston-Salem, NC USA

**Keywords:** Orthopedic oncology, Radiology report triage, Metastatic bone disease, Clinical decision support, Natural language processing

## Abstract

Delays in the referral of patients with suspected metastatic bone disease (MBD) from radiology reports represent a critical challenge that can negatively impact patient outcomes. The conventional manual review process is often a significant bottleneck for triaging, leading to prolonged diagnostic timelines. We developed and validated SHIELD, an automated AI framework designed to accelerate and improve the accuracy of MBD referrals. We fine-tuned a RadBERT-RoBERTa model on a decade of radiology reports (*N* = 245 patients) from two academic medical centers to classify reports into three tiers: “No Referral,” “Referral,” and “Referral/High Risk.” To ensure clinical utility and transparency, SHIELD incorporates a Large Language Model to generate natural-language explanations for its classifications designed to support clinical interpretability and auditability. SHIELD demonstrated high performance on a hold-out test set. It achieved 100% accuracy and an Area Under the Curve (AUC) of 1.00 in the primary binary task of distinguishing referral from non-referral cases. In the more granular three-class task, the model achieved an overall accuracy of 90%, with near-perfect performance in identifying “No Referral” reports (F1-score: 100%). Critically, the model operated in a clinically “fail-safe” manner, never misclassifying a high-risk case as requiring no referral. A retrospective timeline analysis revealed that SHIELD can reduce the referral period from a conventional average of 109.6 days to a computational time of 1–3 min. Proposed work provides high accuracy with a sophisticated explainability component using a large language model. Thus, SHIELD framework is a robust, explainable, and autonomous solution for triaging radiology reports. By drastically reducing administrative and diagnostic delays, it has the potential to significantly accelerate the clinical workflow, ensure timely specialist consultation, and ultimately improve the standard of care for patients with suspected MBD.

**Supplementary Information:**

The online version contains supplementary material available at 10.1007/s10916-026-02373-6.

## Introduction

Metastatic bone disease represents a significant complication arising from advanced malignancies. In the United States, it is estimated that approximately 300,000 adults are living with MBD [1]. Although advancements in systemic therapies for various cancers have improved patient longevity, the prognosis after developing bone metastases is variable, with a wide range of survival rates depending on the primary type of cancer [2]. For example, one-year survival after a bone metastasis diagnosis can range from as high as 51% for breast cancer to as low as 10% for lung cancer [3]. The management of bone metastases also has significant implications for healthcare expenditures. Early intervention for patients with MBD has been demonstrated to reduce patient morbidity and overall healthcare costs, which are estimated to exceed $12.6 billion annually in the United States [4].

Despite the urgent nature of MBD, many patients are not referred to an orthopedic provider until after a pathologic fracture has occurred, leading to a missed opportunity for prophylactic stabilization [5]. A patient’s initial diagnosis of cancer or MBD can be overwhelming for patients and caregivers, with multiple competing priorities occurring at the same time. Knowledge gaps from referring providers on the acuity of impending fractures based upon location, size, or extent of a bone lesion can lead to disparate referral patterns. Access to musculoskeletal experts can also lead to delays in referrals to the treating surgeon. Other sites of metastasis can influence prioritization of referrals and subspecialty appointments, and unintended delays in orthopaedic referrals may occur as a result. This delay underscores the need for improved and standardized referral pathways to orthopedic oncology to enable timely intervention and optimize patient outcomes [6, 7]. While Mirels’ score [8] is a widely used tool within orthopedics to assess fracture risk based on radiographic and clinical features, its adoption outside of the specialty is limited. Furthermore, its low specificity (35%) creates a risk of overtreatment [9]. Referrals to orthopedic specialists are often based on subjective clinical judgment, non-standardized imaging terminology, or non-specific symptoms such as pain, leaving the interpretation and decision to refer largely at the discretion of the non-specialist provider.

Recent advances in artificial intelligence (AI) offer promising solutions to expedite the referral process and reduce the workload of medical experts [10, 11]. For instance, Vergara et al.[12] developed an AI model for gatekeeping referrals from primary to specialized care, which achieved moderate accuracy in distinguishing between authorized referrals, outperforming human gatekeepers by nearly 20% primarily due to higher specificity. Another study demonstrated that incorporating natural language processing (NLP)-extracted qualitative data from referral letters significantly increases the accuracy of machine learning (ML) models by up to 19.5% for triaging patients with low back pain to appropriate interventions, despite the overall model accuracy remaining low for clinical application [13]. The ARCHERY project also utilized NLP with a clinically based large language model (LLM) on free-text radiology reports to predict patient selection for total hip and knee arthroplasties, demonstrating promising potential for hip arthroplasty but not for knee arthroplasty. Their work highlighted the importance of further model testing and training for new clinical cohorts [14].

LLMs being at the forefront of AI have enabled the processing of vast data corpora for various downstream tasks such as classification, analysis, and prediction [15–17]. The promise of these transformer-based architectures has spurred interest in exploring even larger models, often containing billions of parameters. In the biomedical domain, researchers have developed specialized transformer models like BioBERT [18] and PubMedBERT [19] (each comprising 110 million parameters) by training them on biomedical literature from PubMed. Furthermore, clinically specific language models such as RadBERT have been adapted for the field of radiology [20]. These models have demonstrated strong performance in extracting relevant information from free-text radiology reports and have shown potential in predicting the need for surgical interventions based on the language used in imaging reports.

The growing capabilities of AI in healthcare are accompanied by a corresponding scepticism regarding its adoption. A recent study investigated how explainable artificial intelligence (XAI) influences clinicians’ trust in AI applications, addressing the challenge of fostering appropriate reliance on AI’s often “black box” nature [21]. The majority of studies included suggest that XAI has the potential to enhance clinicians’ trust in AI recommendations. However, complex or contradictory explanations can undermine this trust, whereas excessive trust in incorrect AI advice can adversely impact clinical accuracy [22]. This highlights a pressing need for XAI solutions that provide clear, understandable, and reliable explanations, especially due to ethical and regulatory concerns in healthcare [23, 24]. In safety-critical triage workflows, automated recommendations must be transparent and auditable to gain clinician trust and to support accountability in referral decisions. Providing clear, report-grounded explanations allows clinicians to rapidly understand why a case was prioritized, facilitating adoption of AI-assisted triage while preserving clinical oversight.

Despite advancements in the application of AI in healthcare and the associated challenges of explainability, very limited research has specifically addressed the problem of delayed referrals for metastatic bone disease. The overall goal of this study was to introduce **SHIELD** (Skeletal Health Intelligence and Early Lesion Detection), an AI framework designed to prevent late referrals by providing accurate classifications and offering appropriate explainability to aid medical experts in their decision-making. Our study had three aims. The *primary aim* of this work was to (1) develop an AI framework (Fig. 2) to classify radiology reports into three distinct referral categories: no referral needed, referral recommended, and referral/high-risk. This was achieved by fine-tuning the RadBERT-RoBERTa-4m^20^ model for MBD referral classification using a decade of data (January 2014 – May 2025) from two affiliated academic medical centers. RadBERT-RoBERTa-4m [20] is a transformer-based language model developed for radiology and clinical NLP. A *secondary aim* was to (2) provide explanations for the framework’s triaging decisions, thereby increasing trust and adoption among medical providers. We utilized the Llama-3.1-8B-Instruct, Meta’s instruction-tuned language model that was released in July 2024, to accomplish this task. Our *final aim* was to (3) validate the explainability of the proposed framework and assess the overlap between the terminology used by the AI and a predefined set of terms established by orthopedic experts.

## Methods

### Cohort Selection, Radiology Report Review, and Annotation by Clinical Experts

Following institutional review board approval (IRB00127840), we conducted a retrospective review of all patients with a diagnosis of secondary neoplasm of bone treated at two affiliated academic medical centers over a 10-year period (January 2014 – May 2025) by analyzing 1404 Electronic Health Records (EHR). Patients were identified using ICD codes corresponding to secondary malignant neoplasm of bone (C79.51). Clinical documentation and radiology reports from both plain and advanced imaging modalities (PET, CT, MRI, and radiographs) were reviewed.

**Fig. 1:**
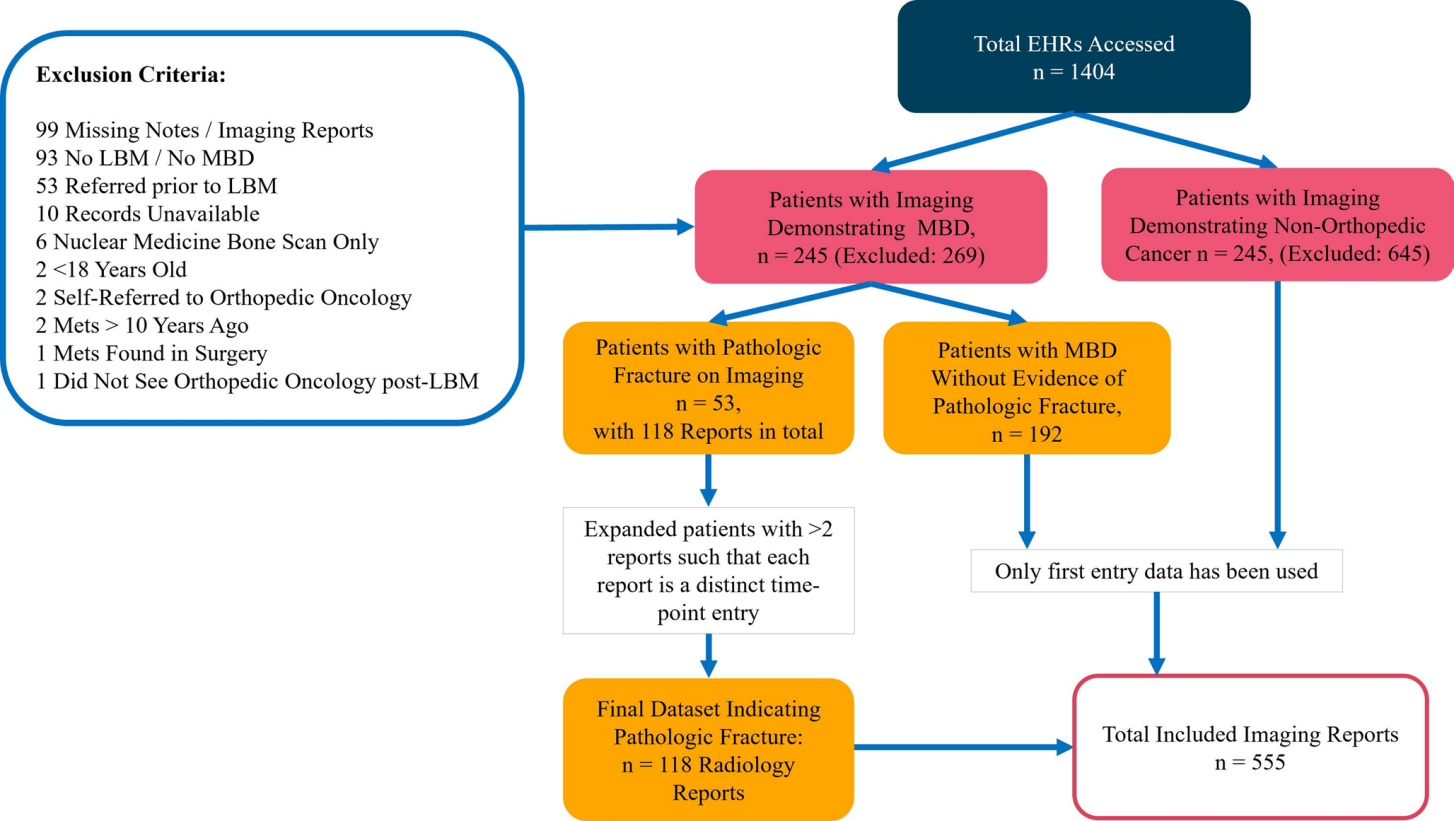
Flow diagram of radiology report selection and preprocessing for SHIELD-based referral classification analysis

Patients were included if they were ≥ 18 years of age at the time of MBD diagnosis and had evidence of osseous metastases to the appendicular long bones, defined as the *femur*, *fibula*, *tibia*, *humerus*, *radius*, or *ulna*. Patients were excluded if they were referred to orthopedic oncology prior to evidence of long-bone metastases (LBM) or were never referred to orthopedic oncology, had their initial imaging dated before January 2014, had only axial or non-LBM, were under 18 years of age at diagnosis, or had incomplete or inaccessible imaging and medical records. From an initial cohort of 514 patients with secondary neoplasm of bone, 245 met full inclusion criteria. A control cohort was generated by screening adult patients with any cancer diagnosis over the same 10-year period who had undergone at least one imaging study (PET, CT, MRI, and radiograph) indicating cancer at either institution. Patients were excluded from this control cohort if they had any mention of osseous metastases in the appendicular skeleton, a diagnosis of primary bone tumor, were under 18 years old, or lacked complete medical documentation. Of the 890 screened patients, 245 met the inclusion criteria for the control group. The cohort selection process is summarized in Fig. 1.

Data was de-identified and collected using REDCap, a secure, web-based platform. Patient demographics, such as age, sex, race/ethnicity, comorbidities, and primary cancer type, were recorded and listed in Table 1. For the MBD cohort, the timeline from first imaging mention of metastasis to referral to orthopedic oncology was documented, along with prior treatments including chemotherapy and radiation. Raw radiology reports were obtained for all relevant imaging studies from the date of the first long bone metastatic lesion to the date of referral. Reports were then analyzed for descriptive language using a list of predefined terms indicative of MBD or SREs. This list of terms was curated a priori by an orthopedic oncologist to avoid bias during data extraction. Specific lesion descriptors, signs of impending fracture, and evidence of progression were recorded. Class 1 (“Referral”) was defined as metastatic long-bone lesions requiring orthopedic oncology evaluation without explicit imaging evidence of an imminent or established fracture. In turn, Class 2 (“Referral/High Risk”) was defined by imaging evidence of a pathologic fracture or descriptors suggestive of impending fracture or structural compromise (e.g., pathologic fracture, cortical breakthrough/destruction, marked lytic lesion with cortical thinning, progression with instability), particularly in weight-bearing bones.

**Table 1:** Participant demographic information based on 245 patients with metastatic bone disease (MBD) including location of primary malignancy and complete trained healthcare professional data

Characteristic	Patients, No. (%)	
Age	Mean = 62 years, (SD = 11.5)	
Sex^a^		
Female	138 (56.3%)	
Male	107 (43.7%)	
Race^a^		
White	167 (61.2%)	
Black or African American	43 (17.6%)	
Asian	3 (1.2%)	
Other	11 (4.5%)	
Ethnicity^a^		
Hispanic or Latino	8 (3.3%)	
Not Hispanic or Latino	237 (96.7%)	
Body Mass Index		
<25	77 (31.4%)	
25.0–30.0	69 (28.2%)	
>30	92 (37.6%)	
Comorbidities^b^		
COPD	16 (6.5%)	
Diabetes	48 (19.6%)	
Hypercholesterolemia	19 (7.8%)	
Hyperlipidemia	53 (21.6%)	
Hypertension	121 (49.4%)	
Osteoporosis	10 (4.1%)	
Renal Insufficiency	12 (4.9%)	
None	81 (33.1%)	
Primary Malignancy Type^c^	Patients with MBD, No. (%)	Patients without MBD, No. (%)
Breast	84 (34.3%)	14 (5.7%)
Kidney	41 (16.7%)	5 (2.0%)
Lung	42 (17.1%)	59 (24.1%)
Lymphoma	1 (0.4%)	22 (9.0%)
Multiple Myeloma	8 (3.3%)	1 (0.4%)
Other^d^	50 (20.4%)	129 (52.7%)
Prostate	17 (6.9%)	14 (5.7%)
Thyroid	2 (0.8%)	1 (0.4%)

a Data on patient demographic characteristics, including age, sex, race, and ethnicity, were collected from the electronic health records at two academic institutions and classified by standardized categories as defined by the investigators

b Indicates selected pertinent comorbidities present at time of first imaging demonstrating MBD

c Data on patient characteristics, including location of primary malignancy, were collected from the EHR at two academic institutions and classified by standardized categories as defined by the investigators

d Includes primary malignancies outside the seven most common cancers known to metastasize to bone

### Data Preprocessing

We curated a dataset of 555 radiology reports annotated by clinical experts into three referral classes: 0 = No Referral, 1 = Referral, and 2 = Referral/High Risk. To ensure a robust evaluation, the dataset was split into a stratified hold-out test set (15%) and a train/validation pool (85%). The stratification preserved class balance across subsets. Randomization was fixed for reproducibility. We trained the ‘No Referral’ and ‘Referral’ classes using only radiology reports from the initial patient visit. In contrast, for the ‘Referral/High Risk’ class, due to the scarcity of the data, we augmented the dataset by treating reports from all of a patient’s visits as distinct data samples. To prevent patient-level information leakage and ensure a clinically realistic evaluation scenario, all data partitioning and cross-validation were performed with strict patient-level separation enforced. In addition, a retrospective analysis of the source reports was performed to avoid label leakage. Only 6.5% of reports explicitly recommended an orthopedic referral (Table 2). The vast majority of reports (72.2%) contained no specific next step recommendation or instead suggested further imaging or clinical correlation.

**Table 2:** Distribution of radiologist-recommended next steps at first imaging suggestive of metastatic bone disease (MBD)

Radiologist-Recommended Next Steps Following Initial Imaging Suggestive of MBD	Count
No Recommendation	177
Suggests Further Imaging	34
Refer to Orthopedic Oncology	16
Refer to Radiation Oncology	1
Repeat Imaging	1
Other	16
Biopsy/Tissue Sample/Pathology	3
Correlate Clinically	6
Correlate with Bone Scan/Other Imaging	5
Follow-Up	2

We applied a sliding-window tokenization strategy using the RadBERT tokenizer, as many reports exceeded the model’s maximum input length. Each report was split into overlapping segments with a maximum length of 512 tokens and a stride of 256 tokens. This approach enabled overlapping token spans and multi-window representation per report while maintaining compatibility with the pre-trained transformer backbone. Tokenization was performed after the data split to prevent leakage between sets. Each segment inherited the label of its original report, producing a window-level dataset for model training and evaluation.

### SHIELD’s Model Architecture for Radiology Report Classification

**Fig. 2:**
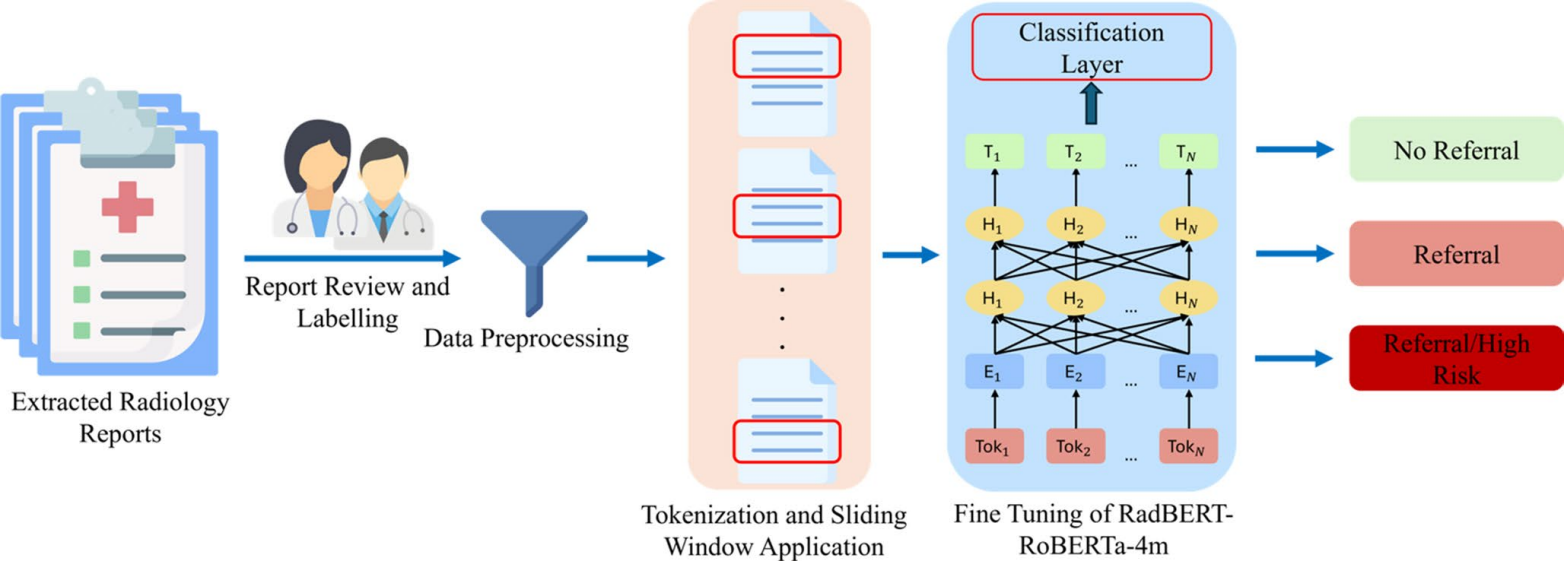
Schematic of the SHIELD’s AI pipeline for automated classification of radiology reports. The workflow begins with the extraction of radiology reports, which are then reviewed and labeled by clinicians. Following data preprocessing, the text is tokenized, and a sliding window technique is applied to create manageable input sequences. These sequences are then used to fine-tune the RadBERT-RoBERTa-4m model. Finally, the model's classification layer categorizes each report into one of three classes: "No Referral," "Referral," or "Referral/High Risk

The proposed classification model is based on the RadBERT-RoBERTa-4m [20] transformer architecture fine-tuned for three-class classes (Fig. 2). The model takes tokenized windows of radiology reports as input. Each window is encoded by the pretrained RadBERT encoder to produce contextual embeddings. The [CLS] token representation is passed through a classification head consisting of a linear layer to generate logits for the three output classes. Cross-entropy loss is used for training.

A 5-fold stratified cross-validation was performed on the train/validation set, maintaining label distribution in each fold. During each fold iteration, tokenized train and validation windows were generated independently. After model inference, window-level predictions were aggregated back to report-level predictions using two schemes:

- Majority vote of the predicted classes across all windows for a report.
- Mean probability aggregation, where class probabilities were averaged across windows and the class with the highest mean probability was selected.

Training was conducted using the Hugging Face Trainer API with the following settings:

- Learning rate: 2e-5.
- Batch size: 4 for both training and evaluation.
- Number of epochs: 8.
- Evaluation and model checkpoint saving at each epoch.
- Best model selection based on validation macro F1-score.
- Logging every 10 steps.

To evaluate generalization, each fold’s model was tested on the same unseen hold-out test set. This set’s tokenization and window-counts were precomputed once to ensure consistent evaluation across folds. Final ensemble predictions were derived by averaging predicted probabilities across all five folds. During inference, the model generates logits for each window, which are converted to probabilities using softmax. These probabilities are then aggregated per report using the two methods described earlier.

To enhance performance and stability:

- We performed model ensembling by averaging predicted class probabilities from all five folds.
- Aggregated predictions were evaluated using both **majority voting** and **mean-max probability**, and both strategies were benchmarked on the hold-out test set.
- Receiver Operating Characteristic (ROC) curve analysis was performed for two key binary tasks: distinguishing “No Referral” from “Referral” and “Referral/High Risk” combined, and distinguishing “Referral” from “Referral/High Risk”, yielding interpretable AUC values to quantify discriminative performance.
- Final outputs included per-class performance metrics (mean ± std across folds), confusion matrices, and ROC curve plots.

Proposed architecture leveraged domain-specific pretraining, sliding-window augmentation, cross-validated ensembling, and robust evaluation strategies to deliver reliable and interpretable triage predictions from unstructured radiology narratives. To benchmark SHIELD’s performance, a baseline comparison was conducted using Gnull [39], a large clinical language model. Both models were evaluated using the same stratified 5-fold cross-validation and hold-out test set. Those results are presented in the Supplementary Material (Fig. S1).

### Generating Interpretable Explanations with SHIELD’s Generative AI

The explainability component of SHIELD was designed to support clinical interpretability and auditability, rather than classical feature attribution or post hoc model introspection. Specifically, the goal was to assess whether large language model generated rationales align with clinician reasoning patterns commonly used during orthopedic oncology referral triage, rather than to explain the internal mechanics of the RadBERT-Roberta based triage classifier. To provide interpretable rationales for each referral prediction, we implemented an automated pipeline using the Llama-3.1-8B-Instruct [25]. The model was hosted on institution-controlled servers to prevent the transmission of protected health information to third-party endpoints, supporting HIPAA-aligned data governance. The Llama-3.1-8B-Instruct was not used for label prediction or model training and had no access to ground truth labels. Instead, it received (1) the original radiology report text and (2) the predicted referral class, and was instructed to extract explicit supporting phrases from the report that justify the assigned triage category. Radiology reports and their predicted class labels (No Referral, Referral, Referral/High Risk) obtained using our fine-tuned UCSD-VA-health/RadBERT-RoBERTa-4m [20] model were stored in a structured CSV file and processed sequentially. For each case, the radiology report text and its predicted label were combined into a class-conditioned prompt. The prompts explicitly requested the model to identify and explain the linguistic or diagnostic features supporting the assigned decision. No few-shot learning approach was employed, and no example prompts were provided to the model. The following prompts were used:

- **Class 0 (No Referral)**:
  - “Identify and explain the terminology or findings that would support **NOT referring** this patient to the Orthopedic Oncology department.”
- **Class 1 (Referral)**:
  - “Identify and explain the key terminology or findings that would indicate the patient **SHOULD be referred** to the Orthopedic Oncology department.”
- **Class 2 (Referral/High Risk)**:
  - “Identify and explain the specific terminology or findings that suggest the patient should be referred to the Orthopedic Oncology department **due to risk of pathological fracture (emergency).**”

**Fig. 3:**
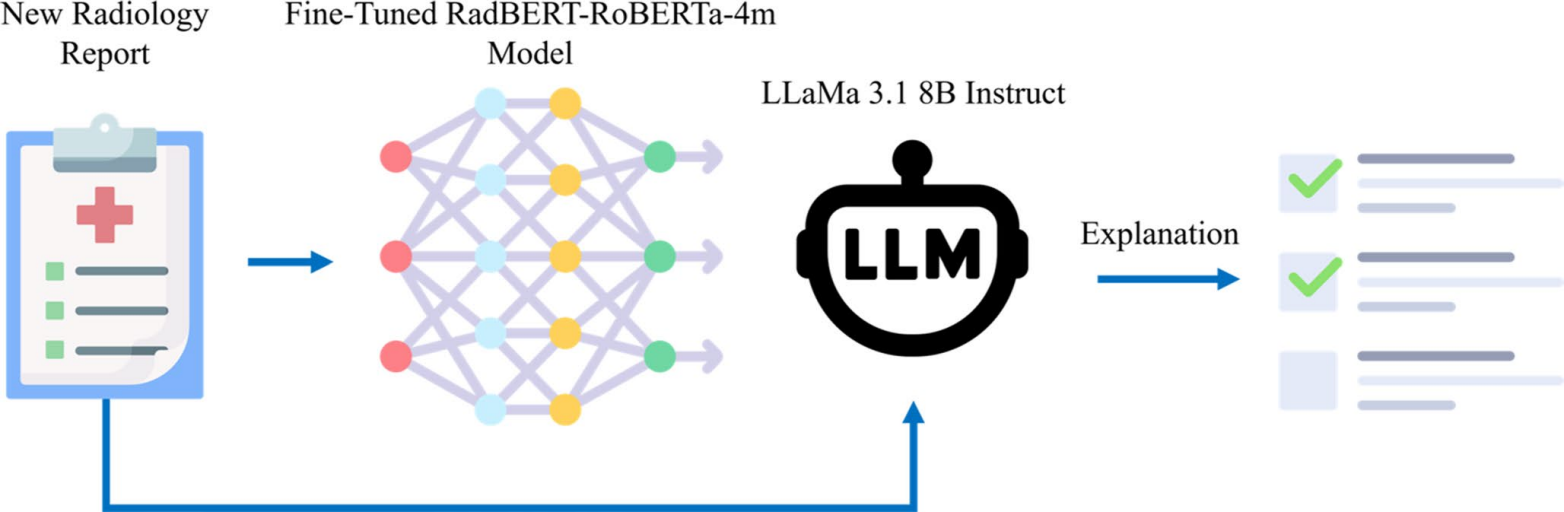
The explainable AI architecture of the SHIELD framework.A new radiology report, along with its classification label (e.g.,"Referral/High Risk") as determined by the classification model, is provided as input to the LLAMA 3.1 8B Instruct model. The LLM then processes this information to generate a natural-language explanation, outlining the key findings and clinical reasoning that led to the initial classification, thereby providing transparency for medical experts

The radiology report text was embedded within this prompt, and the model was queried to generate free-text explanations. Each generation was constrained to a maximum of 1024 tokens with a low sampling temperature (0.3) to encourage concise and reproducible outputs. The resulting explanations were appended to the original dataset as an additional column and exported as a new CSV file for subsequent analysis. This procedure is depicted in Fig. 3 and ensures that every report–prediction pair is accompanied by a standardized, model-generated explanation, enabling a systematic review of the linguistic cues underlying the automated decisions.

### Comparative Analysis of Clinician and LLM-Generated Terminology

To systematically compare terminology used by clinicians with that generated by a large language model (LLM) for orthopedic oncology referral, we implemented a three-phase computational analysis pipeline. The input consisted of two curated term lists: (i) unique medical terms provided by an expert orthopedist (Table S1), which two medical students used to sort radiology reports into three classes and label them, and (ii) a text-based explanation of the decision generated by the LLM, which the medical students filtered out to keep the main terms only.

#### Phase 1: Preprocessing

All terms were normalized to lowercase, stripped of punctuation, tokenized, and lemmatized using the Natural Language Toolkit (NLTK). This ensured consistent lexical forms across both datasets (e.g., “lesions” and “lesion” were reduced to a common root form).

#### Phase 2: Lexical (Set-Based) Analysis

We applied set-theoretic comparisons to quantify overlap and exclusivity between clinician and LLM-derived terminology. Terms were categorized into three groups: shared terms, clinician-only terms (missed by the LLM), and LLM-only terms (absent from clinician usage). The lexical overlap was visualized using a Venn diagram, enabling rapid assessment of concordance and divergence.

#### Phase 3: Semantic and Conceptual Analysis

To capture conceptual similarities beyond exact word matches, we employed a BioBERT (dmis-lab/biobert-v1.1) model to generate vector embeddings of clinician-only and LLM-only terms [26, 27]. Cosine similarity was computed between all term pairs, producing a similarity matrix. This enabled identification of LLM terms that were semantically aligned with clinician terms despite lexical differences. Heatmaps were generated to display the 30 most descriptive term pairs. Furthermore, similarity categories were defined as *High (likely synonyms*,* score > 0.9)*, *Moderate (related concepts*,* score 0.75–0.9)*, or *Low (likely unrelated*,* score < 0.75)*. Results were exported to a CSV file for detailed inspection (Table S2).

This multi-phase pipeline provided both lexical and semantic perspectives on the relationship between clinician-reported and LLM-generated terminology, enabling systematic identification of overlaps, mismatches, and conceptually related terms.

## Results

### Classification Performance

The triaging performance of the proposed SHIELD model was evaluated on the hold-out test set, with performance metrics averaged across a 5-fold cross-validation. Although the majority vote and the mean probability aggregation methods showed very close results, we will only present the latter.

The model demonstrated strong overall performance across the three-class referral triage task (Table 3). The highest performance was observed for the No Referral class, with perfect precision, recall, and F1-score (1.00 ± 0.00), reflecting consistent identification of reports without clinically actionable findings. Performance for the Referral class was similarly robust, with a recall of 0.86 ± 0.03 and an F1-score of 0.85 ± 0.02, indicating reliable detection of metastatic long-bone disease requiring orthopedic oncology evaluation.

**Table 3:** Cross-validated classification performance of the AI model. This table summarizes the performance of the final model on the hold-out test dataset. The reported metrics of precision, recall, and F1-score averaged over five validation folds. Predictions for each report were aggregated by calculating the mean of the maximum probabilities across all text segments. All performance metrics are presented as the mean ± standard deviation

Classes	Precision	Recall	F1-score	Support
No Referral	1.00 ± 0.00	1.00 ± 0.00	1.00 ± 0.00	37
Referral	0.85 ± 0.02	0.86 ± 0.03	0.85 ± 0.02	29
Referral/High Risk	0.77 ± 0.04	0.74 ± 0.05	0.76 ± 0.03	18
Overall Accuracy			0.90 ± 0.01	84

Distinguishing between standard “Referral” and urgent referrals (Referral/High Risk) proved relatively challenging aspect of model performance. For the Referral/High Risk (fracture risk) class, the model achieved a recall of 0.74 ± 0.05, an F1-score of 0.76 ± 0.03, and a precision of 0.77 ± 0.04. No urgent fracture-risk cases were misclassified as No Referral, preserving the safety-oriented design of the triage framework. Overall classification accuracy across all classes was 0.90 ± 0.01.

**Fig. 4:**
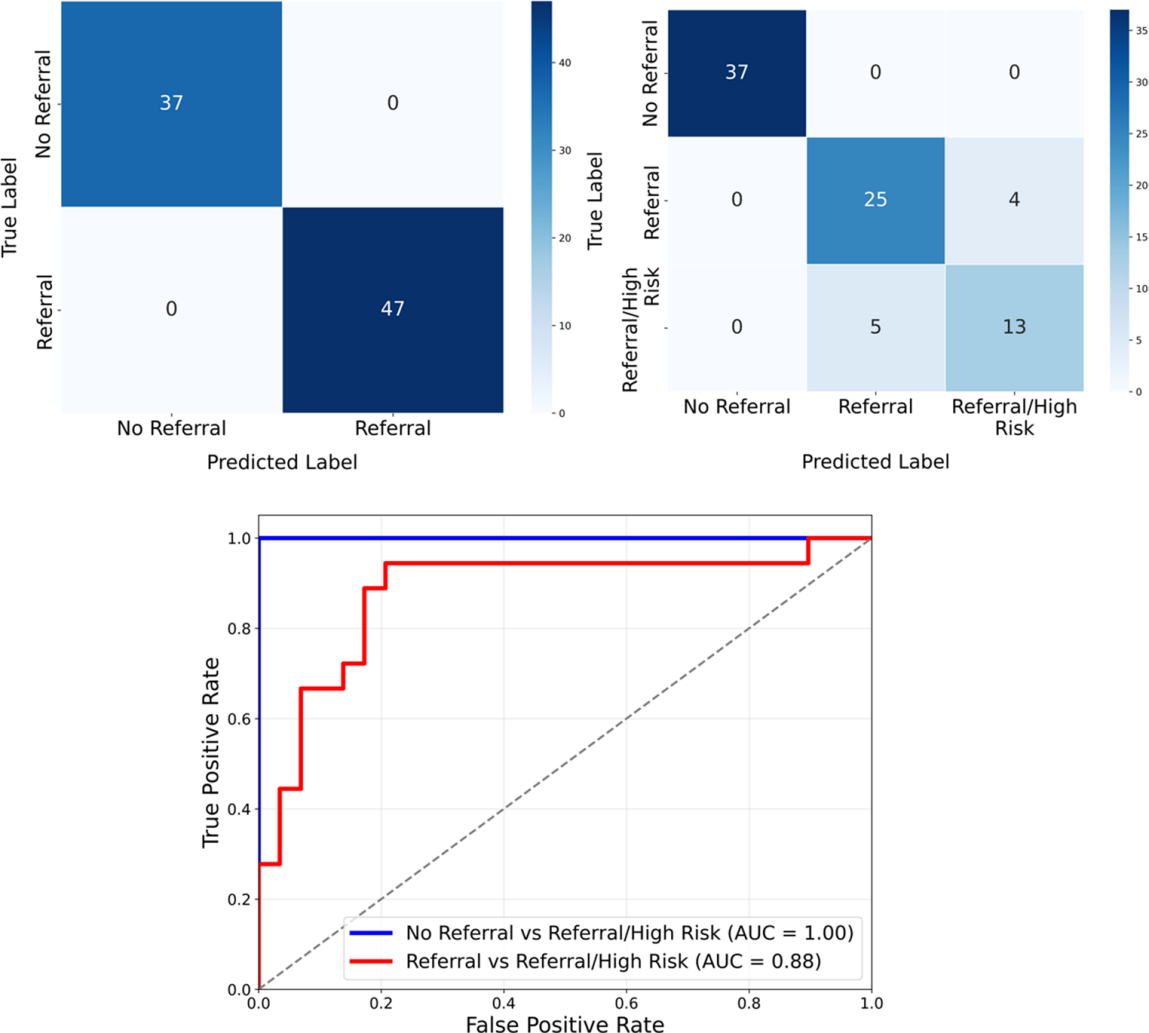
Granular analysis of classification performance using confusion matrices and ROC curves. The figure displays the prediction accuracy of the ensemble model on the hold-out test data. (**a**) The binary confusion matrix demonstrates perfect performance in the primary task of identifying the need for a referral. (**b**) The three-class confusion matrix provides a detailed view, showing that misclassifications are confined to the referral subtypes, where some “Referral/High Risk” cases were classified as standard “Referral.” (**c**) ROC curves for the ensemble model evaluated on the hold-out test set. The blue line represents the perfect separation (AUC = 1.00) between “No Referral” and all referral categories combined. The red line shows excellent discrimination (AUC = 0.88) when distinguishing between “Referral” and “Referral/High Risk” reports

To further visualize the classification performance, confusion matrices for both binary and multi-class scenarios were generated from the ensemble model’s predictions on the hold-out test set. The first matrix (Fig. 4(a)) illustrates the model’s performance on the primary binary task of distinguishing reports that require a referral from those that do not. In this scenario, the “Referral” and “Referral/High Risk” classes were consolidated into a single “Referral” category. The model achieved perfect classification, correctly identifying all 37 “No Referral” cases and all 47 “Referral” cases. This result underscores the model’s exceptional capability to reliably determine whether a clinical follow-up is necessary.

The second matrix (Fig. 4(b)) provides a more granular, three-class analysis. All No Referral cases (37/37) were correctly classified, with no misclassification into referral categories. Among Referral cases, 25 of 29 reports were correctly identified, while 4 were classified as Referral/High Risk. For the Referral/High Risk class, 13 of 18 reports were correctly classified, with the remaining 5 cases classified as Referral. Notably, no Urgent Referral cases were misclassified as No Referral, preserving separation between non-actionable and clinically actionable reports.

The discriminative power of the model was further assessed using Receiver Operating Characteristic (ROC) curve analysis for two key classification scenarios, as shown in Fig. 4(c). For the primary task of distinguishing “No Referral” reports from all referral cases (“Referral” and “Referral/High Risk” combined), the model achieved a perfect Area Under the Curve (AUC) of 1.00. The blue line, representing this task, aligns perfectly with the top-left corner of the plot, indicating a flawless separation between the two groups. In the more granular comparison between Referral and Urgent Referral (fracture risk) cases, the model achieved an AUC of 0.88, reflecting good discrimination between standard and urgent referral categories despite overlapping clinical language.

### Comparison of Conventional vs. SHIELD-Based Referral Timelines

To quantify the potential clinical impact of our AI framework, we compared the referral timelines of the proposed model against the conventional clinical pathway, with the results summarized in Table 4. The analysis revealed that the conventional method is associated with substantial delays, requiring an average of 109.64 ± 286.09 days from the initial imaging report to a patient referral. Additional delays were observed within this period, including an average of 80.57 days from the first documented high-risk descriptor to referral and a diagnostic window spanning 97.50 days between the first and last imaging events prior to referral. In stark contrast, the AI model provides its classification recommendation within 1 to 3 min of processing the initial report. By generating an immediate recommendation from a single data point, the model renders the prolonged, multi-step diagnostic timelines of the conventional pathway obsolete, demonstrating its potential to drastically reduce administrative and clinical delays inherent in the current standard of care.

**Table 4:** Comparison of referral timelines between conventional methods and the proposed AI model

Metric	Conventional Method (days, mean ± SD)	Proposed AI Model (minutes)
Time from Initial Imaging to Referral Decision	109.64 ± 286.09	~ 1–3
Time from First High-Risk Finding to Referral	80.57 ± 256.87	
Diagnostic Period (First to Last Imaging Pre-Referral)	97.50 ± 281.99	

Of the 245 patients evaluated, 124 (50.61%) had undergone at least two imaging visits prior to referral. Additionally, 85 patients (34.69%) experienced delayed referrals, defined as a referral made more than 28 days after the first imaging report indicating potential bone malignancy. The proposed AI solution demonstrated promising results by substantially reducing the referral delay, successfully classifying referrals based solely on the first imaging report for “No Referral” and “Referral” classes.

### Explainable AI

Table 5 presents a side-by-side comparison of an original radiology report accurately classified as Referral/High-Risk and its corresponding AI-generated explanation. Supplementary material (Table S3) holds other examples of Llama-3.1-8B-Instruct model generated explanations.

**Table 5:** An example of an explained radiology report provided by Llama-3.1-8B-Instruct

Original Radiology Report (Referral - High Risk)	AI Explanation (Referral - High Risk)
- CT PELVIS WO CONTRAST (ROUTINE) Status: Final result Study Result Narrative & Impression CT UNINFUSED PELVIS	The patient should be referred to the Orthopedic Oncology department due to the risk of pathological fracture (emergency) because of the following specific terminology and findings in the radiology report:
INDICATION: please image as much of right femur as possible \ eval fracture \ M25.551 Right hip pain COMPARISON: Right hip radiographs from …	1. **Pathological fracture**: The report mentions an “acute pathological subcapital right femoral neck fracture” (CT pelvis) and an “acute subcapital femoral neck fracture” (X-ray thigh/femur). This indicates that the fracture is not a typical traumatic fracture but rather a fracture that occurs due to a pre-existing bone disease or weakness, which is a characteristic of pathological fractures.
TECHNIQUE: Thin-section images of the pelvis were obtained from the data set of the CT chest, abdomen, and pelvis with IV contrast. All CT scans at Wake Forest Baptist Medical Center and Wake Forest Baptist Imaging are performed using dose optimization techniques as appropriate to a performed exam, including but not limited to one or more of the following: automated exposure control, adjustment of the mA and/or kV according to patient size, use of iterative reconstruction technique. In addition, Wake is participating in the ACR Dose Registry program which will further assist us in optimizing patient radiation exposure.	2. **Lytic lesions**: The report describes “mottled appearance of the right femoral head and neck with numerous lytic lesions surrounding the fracture site” (CT pelvis) and “lucencies within the greater trochanter and along the fracture line” (X-ray thigh/femur). Lytic lesions are areas of bone destruction, which can be a sign of metastatic disease or other bone pathology.
ADDITIONAL TECHNIQUE: None. FINDINGS: Acute pathological subcapital right femoral neck fracture with varus and anterior apex angulation at the fracture site. The dominant distal fracture fragment is displaced superiorly. Mottled appearance of the right femoral head and neck with numerous lytic lesions surrounding the fracture site. Subacute, healing pathological fracture through a lytic lesion within the right ischiopubic ramus. Additional lytic lesions involving the anterior aspect of the L4 vertebral body, along the left aspect of the L5 superior endplate, and left ilium concerning for additional foci of osseous metastatic disease.	3. **Metastatic disease**: The report mentions “scattered lytic lesions involving the lower lumbar spine, pelvis, and the right proximal femur concerning for osseous metastatic disease” (CT pelvis). This suggests that the patient may have metastatic disease, which increases the risk of pathological fractures.
Transitional lumbosacral anatomy with right L5-S1 pseudoarticulation. Sclerosis surrounding the bilateral SI joints suggestive of osteitis condensans ilii. IMPRESSION: (1) Acute pathological subcapital right femoral neck fracture with apex varus and anterior angulation and superior displacement of the distal fracture fragment. (2) Scattered lytic lesions involving the lower lumbar spine, pelvis and the right proximal femur concerning for osseous metastatic disease.	4. **Bone pain**: The report mentions “M25.551 Right hip pain” as the indication for the imaging studies. Bone pain can be a symptom of pathological fractures or metastatic disease.
… - XR THIGH/FEMUR RIGHT 2 V PORTABLE Status: Final result Study Result Narrative & Impression X-RAY RIGHT FEMUR (PORTABLE), …	Given these findings, the patient should be referred to the Orthopedic Oncology department for further evaluation and management of the potential pathological fracture and underlying bone disease. The Orthopedic Oncology department can provide specialized care for patients with bone tumors and metastatic disease, and can help to manage the risk of pathological fractures. This is an emergency situation, as the patient is at risk of a pathological fracture, which can be a life-threatening complication. Prompt referral to the Orthopedic Oncology department is necessary to prevent further complications and ensure the patient receives appropriate care.
INDICATION: Hip Fracture eval - marker ball \ M25.551 Right hip pain COMPARISON: None.	
IMPRESSION: 1. Acute subcapital femoral neck fracture with varus alignment and superior displacement of the distal fracture fragment.	
2. Lucencies within the greater trochanter and along the fracture line are nonspecific but raise concern for possible pathologic fracture.	
3. The hip is located.	

**Fig. 5:**
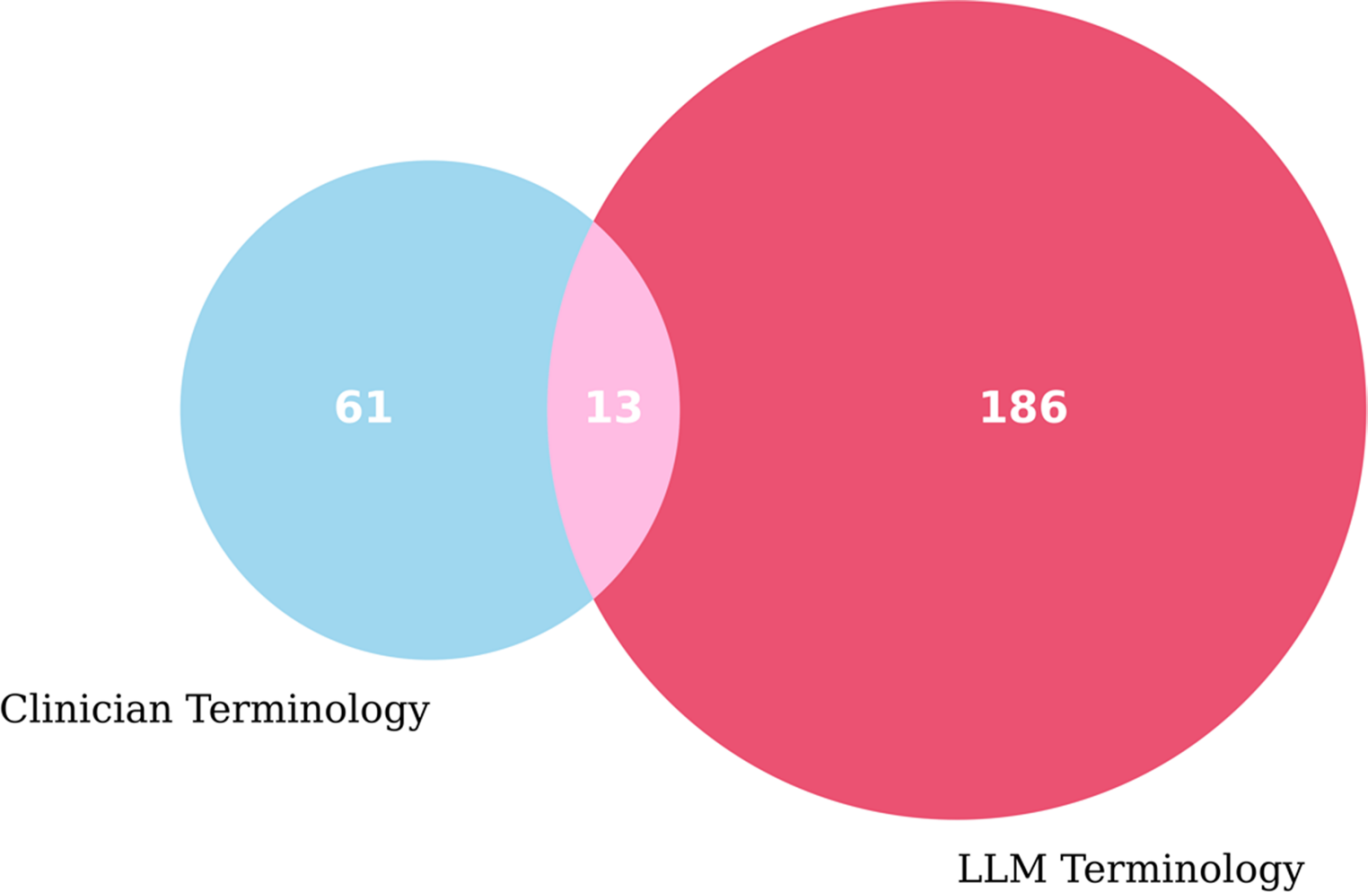
Venn diagram illustrates the overlap between LLM and clinician selected term sets

The complete list of reports explained are available as supplementary material. In our analysis, an expert orthopedic doctor provided 74 unique descriptive terms, while the LLM produced 199 unique terms after careful filtering. The Venn diagram in Fig. 5 illustrates the overlap between these term sets, showing 13 terms that were common to both the expert and the LLM: ‘enhancing lesion’, ‘periosteal reaction’, ‘aggressive appearing’, ‘cortical disruption’, ‘cortical destruction’, ‘expansile’, ‘cortical erosion’, ‘aggressive’, ‘cortical thinning’, ‘t1 hypointense t2 hyperintense’, ‘endosteal scalloping’, ‘lytic lesion’, ‘cortical breakthrough’. Some examples for clinician-only terms were ‘predisposed to pathologic fracture’, ‘lytic change’, ‘increased risk of pathologic fracture’, ‘hypermetabolic osseous disease’, ‘cortical transgression’, ‘concerning for instability’, ‘hypointense’, ‘mixed lytic’, and ‘increased uptake in bone’. In contrast LLM specific terms were more lexically longer like ‘localized pathologic fracture’, ‘sclerotic lesion’, ‘expansile lytic lesion involving the left anterior 5th rib’, ‘fracture’, ‘proximal femur’, ‘proximal right femur’, ‘presence of lytic lesion and sclerotic lesion indicating bone metastasis’, ‘possibility of a primary osseous neoplasm or lymphoma cannot be excluded’, ‘mildly displaced pathologic fracture’, ‘indeterminate lucent lesion’.

**Fig. 6:**
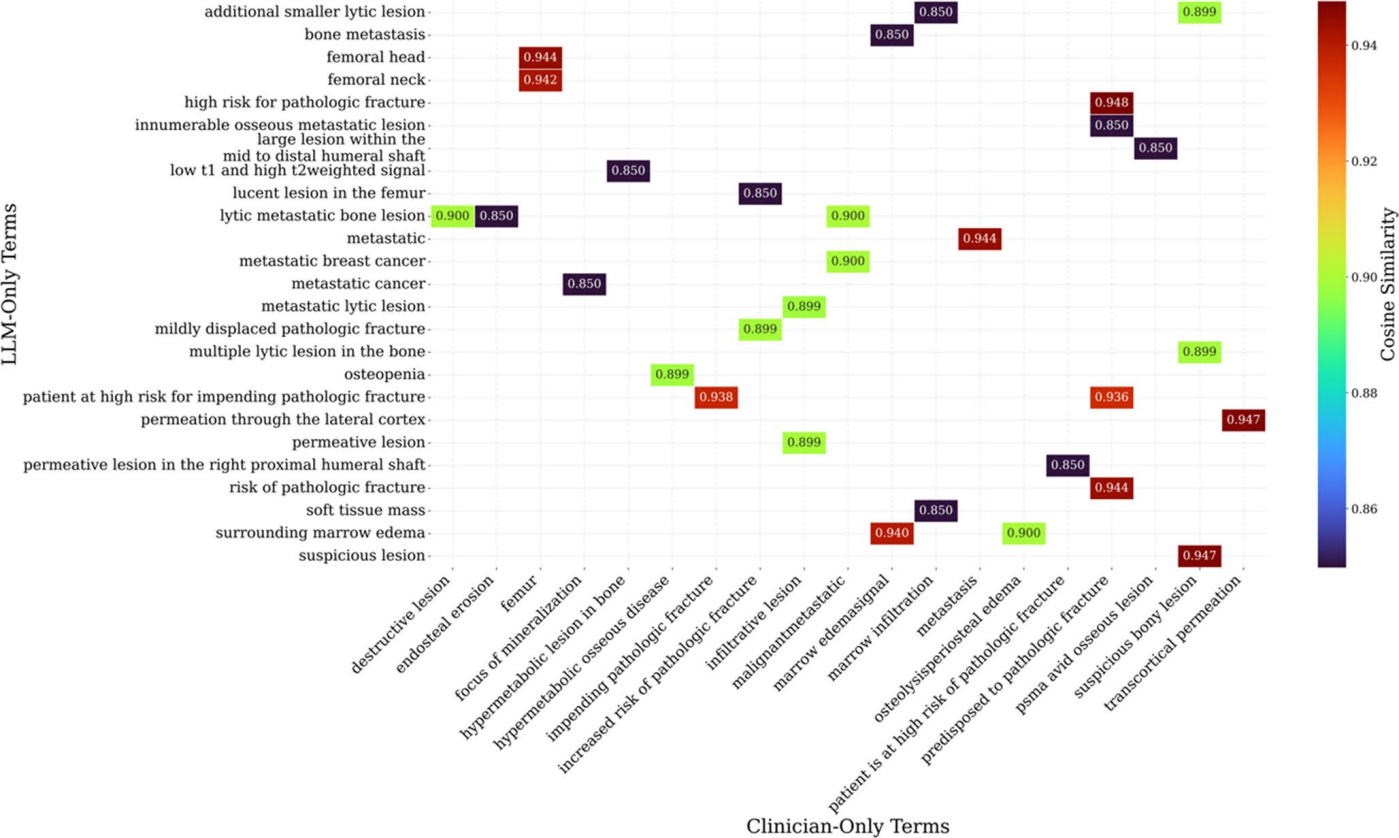
Heatmap showing 30 demonstrative pairs for LLM and Clinician generated terms. By using BioBERT to analyze conceptual relationships, this visualization effectively identifies example pairs of terms that are semantically aligned despite differences in their exact wording. The colored squares indicate the cosine similarity score for a given pair, with warmer, red tones representing higher similarity and cooler, blue tones representing moderate similarity

To evaluate the semantic alignment between the LLM’s generated explanations and standard clinical terminology, we performed a cosine similarity analysis on keywords and phrases that were unique to either the LLM’s output (“LLM-Only”) or the clinician’s lexicon (“Clinician-Only”) demonstrated in Fig. 6. The results, presented in a heatmap, reveal a high degree of semantic congruence between the two vocabularies, even when the exact phrasing differed. Multiple term-pairs exhibited strong conceptual overlap with similarity scores exceeding 0.90. For instance, the LLM-generated phrase “permeation through the lateral cortex” was found to be almost identical in meaning to the clinician’s term “transcortical permeation” (similarity = 0.947). Similarly, the LLM’s more specific “femoral head” and “femoral neck” showed very high similarity (0.944 and 0.942, respectively) to the broader clinician term “femur.” We also observed that the LLM’s descriptive phrase “lytic metastatic bone lesion” was a strong semantic match to the concise clinical term “metastasis” (similarity = 0.900), confirming the model’s ability to capture clinical meaning accurately. Another pair from LLM being “soft tissue mass” with the clinician’s “marrow infiltration” at a score of 0.85 demonstrates a clear example of a lexically different but contextually similar pair. This heatmap shows that the LLM can generate lexically diverse but clinically relevant and semantically equivalent terms when compared to an expert clinician’s vocabulary.

## Discussion

Prior work from our group has demonstrated the growing role of artificial intelligence in orthopedics and oncology. In orthopedics, we applied machine learning to model fracture recovery using gait analysis, offering predictive insights into patient outcomes following lower extremity fractures. Beyond orthopedics, we advanced interpretable AI frameworks for oncology, including [28, 29] deep learning models for tumor classification and cancer recurrence prediction [30, 31]. We also developed multimodal language–vision assistants in pathology, highlighting the feasibility of adapting LLMs for domain-specific interpretation [32]. Collectively, these contributions provide a foundation for the present work, which builds on this trajectory by addressing delayed referrals in metastatic bone disease with both accurate classification and explainable AI-driven explanations. In response to the critical issue of delayed referrals for metastatic bone disease, this study successfully developed and validated a dual-component AI framework of SHIELD designed to both classify and explain findings in radiology reports. Complementing its predictive power, the explainability component of Llama-3.1 model demonstrated its ability to generate clinically coherent rationales.

SHIELD’s classification model demonstrates strong utility as a clinical decision support tool by automating the initial triage of radiology reports in a safety-critical orthopedic oncology workflow. The model achieved perfect performance in identifying No Referral cases (F1-score of 1.00), indicating reliable exclusion of non-actionable reports. This capability is particularly relevant in high-volume clinical environments, where safely filtering out reports without urgent orthopedic implications can substantially reduce clinician workload and allow greater focus on patients requiring intervention. Consistent with this, binary classification between No Referral and any actionable referral (Referral and Referral/High Risk) yielded an AUC of 1.00, demonstrating complete separation between non-actionable and clinically relevant reports in this cohort.

The more clinically nuanced task lies in distinguishing between standard Referral and Referral/High Risk cases, where imaging language often overlaps and urgency is conveyed implicitly rather than explicitly. In this setting, the model achieved a recall of 0.74 and an F1-score of 0.76 for the urgent fracture-risk category, with a precision of 0.77. Importantly, analysis of the confusion matrix shows that no urgent fracture-risk cases were misclassified as No Referral. All misclassifications occurred between the Referral and Referral/High Risk categories, reflecting underestimation of urgency rather than failure to identify clinical relevance. From a patient safety perspective this behavior is highly preferable, as it ensures that all patients at risk for pathologic fracture are flagged for orthopedic evaluation, even if some are initially triaged as standard rather than urgent referrals. The ROC analysis further supports this interpretation. While discrimination between Referral and Referral/High Risk cases (AUC = 0.88) was lower than that for the primary screening task, it still indicates a good separability given the inherent ambiguity in radiology report language regarding fracture risk. These findings suggest that SHIELD is well-suited for use as a first-line triage tool, with future optimization efforts focused on improving sensitivity for urgent fracture-risk cases.

The stark contrast in referral timelines highlights the most profound clinical implication of the SHIELD framework: its potential to dismantle the protracted and inefficient nature of the current diagnostic pathway. Clinical triaging with an average delay of nearly 110 days from initial imaging to referral is not merely slow but is characterized by systemic inefficiencies. Our findings show that over half of patients required multiple imaging visits and more than a third experienced referral delays exceeding a month, underscoring a period of clinical uncertainty and patient anxiety. In direct contrast, the AI model’s ability to generate a reliable classification in minutes from the very first imaging report represents a paradigm shift. It obviates the need for a prolonged “watch-and-wait” period and multiple follow-up scans, which are currently the primary drivers of these delays. By intervening at the earliest possible point, the framework transforms a reactive, multi-step process into a proactive and immediate decision point. This can potentially ensure the journey from initial suspicion to specialist consultation begins without delay.

This investigation demonstrates the framework’s robust capability to process and reinterpret specialized medical language for clinical applications. The key finding is the significant disparity between the low lexical overlap and the high semantic similarity when comparing the LLM’s output to an expert clinician’s terminology. This suggests that the LLM is not merely extracting keywords but is generating a conceptually aligned, and often more descriptive, vocabulary to explain complex radiological findings. The model’s ability to produce lexically diverse yet semantically equivalent terms underscores its potential as a sophisticated tool for clinical communication. By translating dense medical reports into structured and simplified explanations, as exemplified in the case study, the model can serve as a valuable aid in enhancing patient understanding and supporting clinical decision-making.

Our work contributes to the growing body of research on AI-driven clinical triage, sharing the overarching goal of improving upon manual, often inefficient processes, as seen in both emergency [33] and specialist settings. While our framework addresses a specialized referral pathway similar to recent studies in gatekeeping and triage, its classification performance notably surpasses these prior efforts. For instance, the AI gatekeeping model by Vergara et al.[12] achieved an overall accuracy of 71.6% (AUC of 0.765) in a binary referral authorization task across multiple specialties. Similarly, the work by Maarseveen et al.[34] reported an AUC of 0.78 for prioritizing rheumatology referrals, and the system by Abdel-Hafez et al.[35] showed a 53.8% level of agreement for categorizing ENT referrals. A directly comparable study was recently introduced by Sangwon et al.[36], who evaluated three NLP models for automating bone metastases referrals: a rule-based Regular Expression (RegEx) model, GPT-4, and a specialized BERT model (NYUTron). Their findings indicated that the RegEx model often outperformed the more complex LLMs for their specific clinical application, achieving an F1-score of 88.9% during validation. While this work represents a valuable contribution to AI-driven triage, it has notable limitations, such as the lack of a clinically validated timeline comparison and the use of a dataset covering a shorter time frame.

In contrast to these studies, our SHIELD framework demonstrates a significant advance in performance, achieving 100% accuracy in the binary Referral vs. No Referral task and a very good AUC of 0.88 in the more complex three-class problem. Furthermore, a key architectural advantage of our framework is its operational autonomy; following the initial data labeling phase, the model operates independently without requiring real-time clinician interrogation or rule-based adjustments for its predictions. This performance advantage likely stems from our model’s unique design, which enables it to interpret the dense, unstructured narrative of a full radiology report for a specialized oncological pathway. However, the most significant departure remains our framework’s integral dual-component architecture. Where other models focus primarily on the classification task, our work pairs its high accuracy with a sophisticated explainability component using a large language model. This distinction is crucial [37], as our goal is not only to accelerate a workflow but also to build clinician trust and facilitate communication for a considered specialist referral—a focus on interpretability that moves beyond what is presented in related literature.

This study has several limitations. Our dataset consisted of 1,404 EHRs collected over ten years. Furthermore, the scope was restricted to malignancies of the long bones (femur, fibula, tibia, humerus, radius, and ulna). The model’s performance on the ‘Referral/High Risk’ class was impacted by a relatively small number of samples in this category, which presented a challenge for achieving optimal classification accuracy. Although no high-risk cases were labeled ‘No Referral,’ highlighting the preservation of safe screening, downgrading urgency may still delay fracture-prevention pathways. Considering that SHIELD is designed as a triage support tool designed to minimize missed fracture-risk cases; however, urgency downgrades remain clinically meaningful errors and are explicitly tracked, our future work will aim to address these limitations. Subsequent iterations will prioritize sensitivity for class 2 via cost-sensitive loss weighting/focal loss, and an escalation rule based on the predicted probability of class 2 (e.g., flag as urgent if Probability(class 2) exceeds a clinically selected threshold). We also plan to expand the dataset by including a larger number of EHRs and incorporating all sites of skeletal metastasis. Additionally, integrating multimodality by adding image analysis could further enhance the model’s performance, making it an even more reliable tool for medical experts [38]. Collectively, these findings validate the framework’s potential to significantly accelerate the patient referral pathway while fostering trust and adoption among clinicians through transparent and meaningful explanations.

## Conclusion

As the population ages and the prevalence of cancer continues to rise, healthcare systems face increasing demands to efficiently triage patients with metastatic bone disease and identify those at greatest risk for skeletal-related complications. This study presents SHIELD, an AI-enabled radiology report triage framework designed to support timely referral prioritization in orthopedic oncology, with particular emphasis on identifying imaging language suggestive of mechanical instability and pathologic fracture risk.

Rather than serving as a diagnostic or prognostic tool, SHIELD is intended to augment existing clinical workflows by automatically flagging reports that warrant expedited subspecialty evaluation. The results demonstrate that natural language processing can reliably distinguish between reports requiring no referral, standard referral, and urgent referral due to fracture risk, while maintaining a safety-first design that does not dismiss high-risk cases. The inclusion of clinician-aligned, language-based explanations further supports auditability and interpretability in a safety-critical triage context.

## Supplementary Information

Below is the link to the electronic supplementary material.

Supplementary Material 1 (DOCX 1.25 MB)

## Data Availability

The dataset from this study is held securely in coded form Wake Forest University School of Medicine. The full dataset creation plan are available from the authors upon request. The source code for implementing the methods is available at the following repository: https://github.com/CAIR-LAB-WFUSM/SHIELD.git.
