# Supplementary material for: SHIELD: An AI Framework for Skeletal Health Intelligence and Early Lesion Detection to Improve Orthopedic Referrals
Source: J Med Syst. 2026 Apr 13;50(1):51. doi: 10.1007/s10916-026-02373-6 (PMC13076404; doi:10.1007/s10916-026-02373-6)
Supplement: Supplementary file 1 — Supplementary Material 1 (DOCX 1.25 MB) [file 10916_2026_2373_MOESM1_ESM.docx]

**Supplementary Materials**

**Table S1.** Comprehensive list of unique medical terms provided by an expert orthopedist, which two medical students used to sort radiology reports into three classes and label them.

| **Category** | **Subcategory** | **Terms/Descriptors** |
| --- | --- | --- |
| Anatomic | General Bone | Bony, Bone, Osseous |
|  | Upper Extremity | Humerus/ Humeral, Radius, Ulna |
|  | Lower Extremity | Femur/ Femoral, Tibia/ Tibial, Fibula |
| Disease | Metastatic | Metastatic/ metastases, Malignancy/ malignant, Neoplastic |
|  | Pattern | Lytic (hypodense, lucent), Sclerotic (blastic, hyperdense, radiopaque), Mixed (lytic/sclerotic) |
|  | Margins | Well-defined, Ill-defined |
|  | Lesion | PSMA avid osseous lesion, Aggressive appearing, Infiltrative lesion, enhancing lesion FDG avidity/ uptake in (bone), (Increased) SUV max, T1 hypointense T2 hyperintense, Increased uptake in (bone), Hypermetabolic lesion, Hypermetabolic osseous disease, Focus of mineralization, Suspicious bony lesion, Lucency, Expansile, Hyper-enhancing, Hypointense, Destructive |
| Clinical | Bony Concerns | Loss of cortical integrity, Endosteal scalloping, Periosteal reaction, Cortical thinning, Cortical erosion, Cortical destruction, Cortical breakthrough, (Trans)cortical permeation, Osseous destruction, Osteonecrosis, Signal change (marrow), Marrow replacing, Cortical thickening, Marrow infiltration, Cortical narrowing, Endosteal erosion, Cortical discontinuity, Transcortical soft tissue extension, Suspicious for progression of osseous disease |
|  | High Risk | Lesion/ patient is at (high) risk of pathologic fracture, Increased risk of pathologic fracture, impending pathologic fracture, predisposed to pathologic fracture, Loss of cortical integrity, Lytic changes, Lytic lesion, Destructive lesion, Moth-eaten appearance, Mottled, Concerning for instability, Cortical thinning, Cortical erosion, Cortical destruction, Cortical breakthrough, Cortical transgression. Cortical dehiscence, Osseous destruction, Osteolysis, Periosteal edema/ reaction, Periostitis, Endosteal scalloping, Aggressive/ aggressive appearing, Permeative, Possible pathologic fracture, Cortical permeation, Cortical disruption, Cortical loss, Osteonecrosis, Marrow edema/signal, Cortical discontinuity |
|  |  |  |

**Table S2: Cross-Validated Classification Performance of the GatorTron Model.** This table summarizes the performance of the final model on the hold-out test dataset. The reported metrics of precision, recall, and F1-score averaged over five validation folds. Predictions for each report were aggregated by calculating the mean of the maximum probabilities across all text segments. All performance metrics are presented as the mean ± standard deviation.

| **Classes** | **Precision** | **Recall** | **F1-score** | **Support** |
| --- | --- | --- | --- | --- |
| No Referral | 0.99 ± 0.01 | 1.00 ± 0.00 | 0.99 ± 0.01 | 37 |
| Referral | 0.82 ± 0.01 | 0.83 ± 0.02 | 0.82 ± 0.01 | 29 |
| Referral/High Risk | 0.73 ± 0.02 | 0.70 ± 0.03 | 0.72 ± 0.01 | 18 |
| Overall Accuracy |  |  | 0.88 ± 0.01 | 84 |


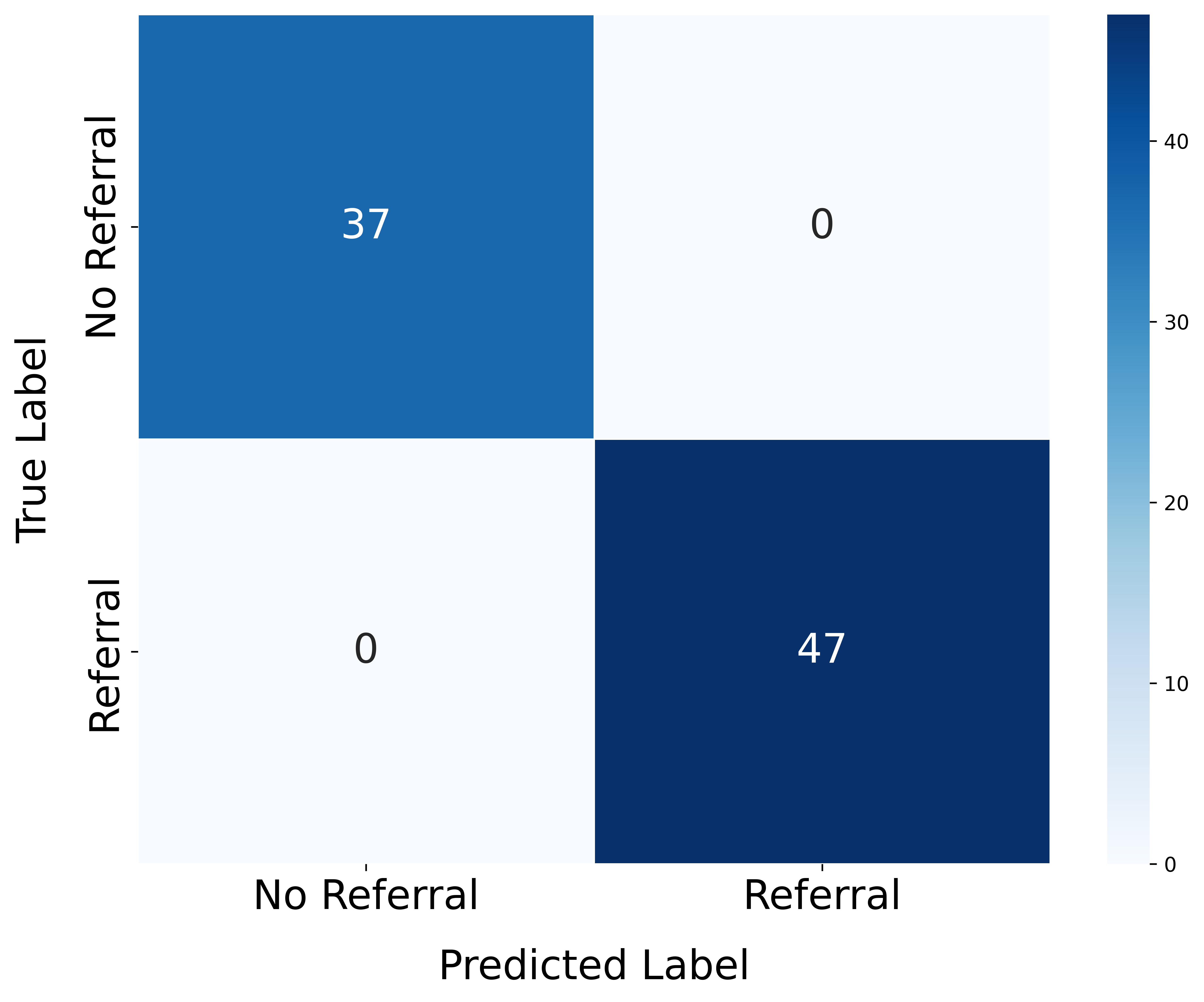

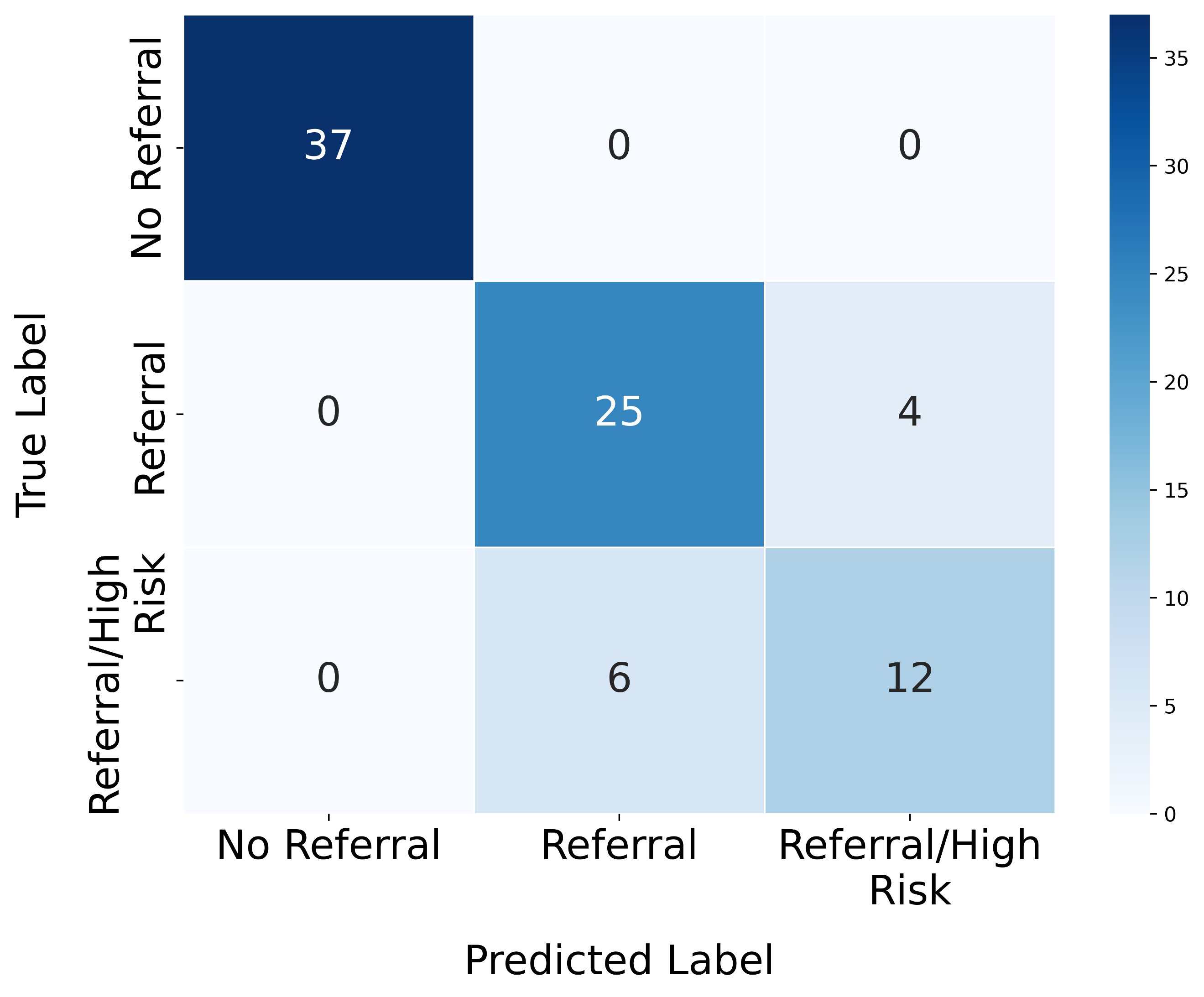


**
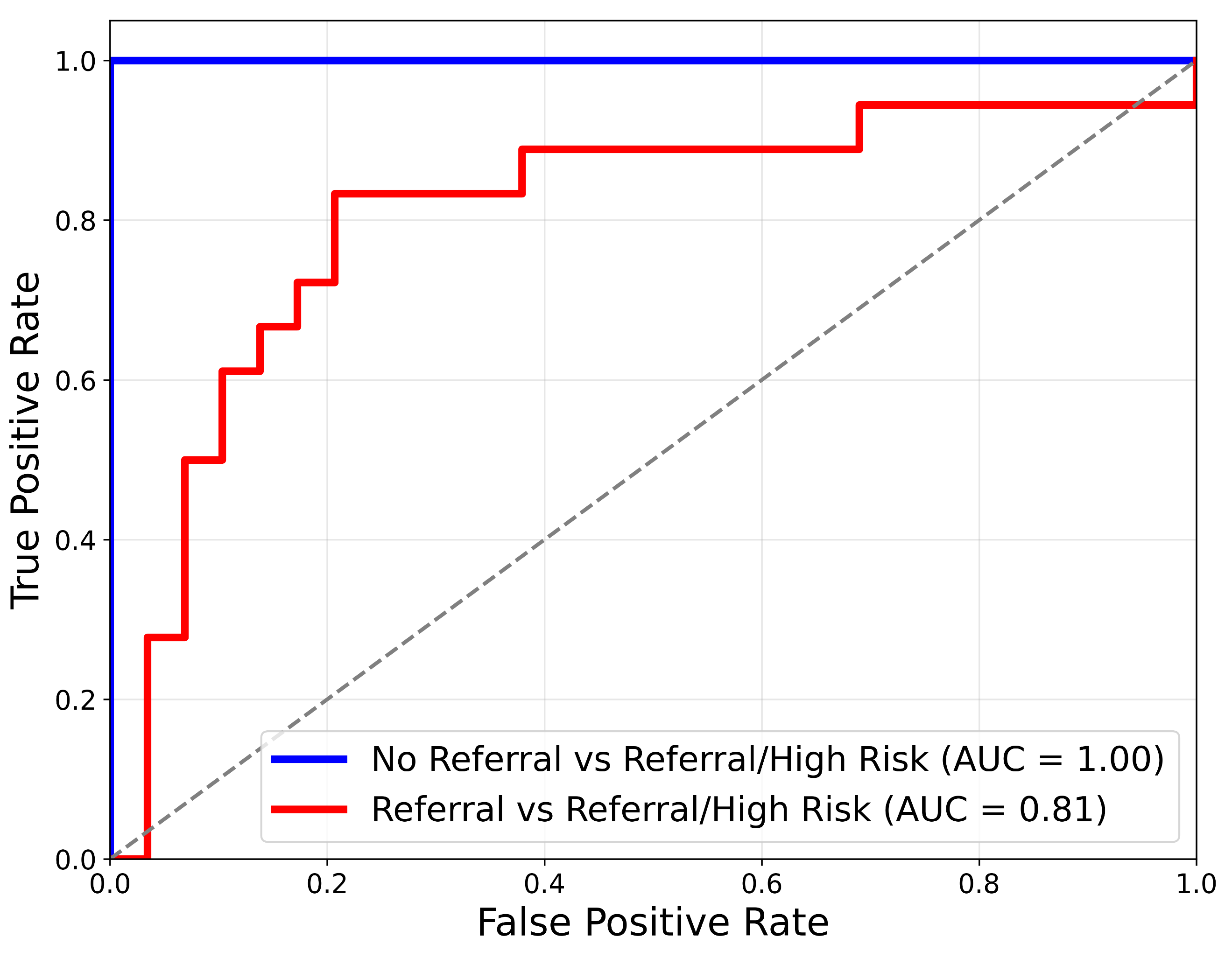
**

**Figure S1.** **Granular Analysis of Classification Performance of GatorTron Model using Confusion Matrices and ROC curves.** The figure shows the ensemble model's prediction accuracy on the hold-out test data. (a) The binary confusion matrix demonstrates perfect performance in the primary task of identifying the need for a referral. (b) The three-class confusion matrix provides a detailed view, showing that misclassifications are confined to the referral subtypes, where some "Referral/High Risk" cases were classified as standard "Referral." (c) ROC curves for the ensemble model evaluated on the hold-out test set. The blue line represents the perfect separation (AUC = 1.00) between "No Referral" and all referral categories combined. The red line shows relatively weaker discrimination (AUC = 0.81) when distinguishing between "Referral" and "Referral/High Risk" reports.

**Table S3.** Demonstrative examples of an explained radiology report provided by Llama-3.1-8B-Instruct. Llama-3.1-8B-Instruct has not carried out the classification but relied on Radbert Roberta model’s decision and the original report for explanations

| **Original Radiology Report  (No Referral)** | **AI Explanation (No Referral)** |
| --- | --- |
| CT Chest Abdomen Pelvis W Contrast Status: Final result Study Result Narrative & Impression CT CHEST ABDOMEN PELVIS W CONTRAST, … INDICATION: Endometrial cancer re-assessment, Malignant neoplasm of corpus uteri, unspecified (CMD) \ C54.9 Malignant neoplasm of corpus uteri, unspecified (CMD) COMPARISON: CT CAP … . Outside CT A/P … TECHNIQUE: CT images of the chest, abdomen, and pelvis were obtained following intravenous administration of iodinated contrast. Conventional axial reconstructions and multiplanar reformatted images were submitted for review. FINDINGS: Thoracic inlet: Within normal limits. Chest wall/Axilla: New nodular soft tissue in the right breast measuring up to 1.7 cm. . Central airways: Patent. Mediastinum/Hila: Small sliding hiatal hernia. No adenopathy. . Heart: Heart size within normal limits. No pericardial effusion. Coronary artery calcifications. Aortic valve calcifications. . Vessels: Aorta normal in caliber. No central pulmonary embolism. Lungs/Pleura: No large airspace consolidation or pleural effusion. Liver: No suspicious focal findings. Gallbladder/Biliary: Cholecystectomy. Spleen: Similar partially calcified hyperattenuating lesion and numerous punctate calcified granulomas. Pancreas: Unremarkable. Adrenals: Unremarkable. Kidneys: Similar left renal cyst. Peritoneum/Mesenteries/Extraperitoneum: No free air. No free fluid or loculated drainable collection. No pathologically enlarged lymph nodes. . Gastrointestinal tract: Redemonstrated large right inguinal defect containing multiple loops of nondilated large and small bowel. No obstruction. Ureters: Unremarkable. Bladder: As below. Reproductive System: Hysterectomy and bilateral salpingo-oophorectomy. Soft tissue mass measuring 3.6 x 4.4 x 5.1 cm (AP x TV x CC) at the anterior margin of the lower anterior margin of the vaginal cuff and is inseparable from the urinary bladder base. No clear invasion into the urinary bladder. Vascular: Aortoiliac atherosclerotic disease. Musculoskeletal: Right hemipelvectomy. No acute displaced fractures. Polyarticular degenerative changes. No aggressive focal bony lesions. IMPRESSION: 1. Soft tissue mass at the lower/anterior margin of the vaginal cuff, inseparable from the base of the urinary bladder, highly concerning for new site of disease. Recommend pelvic MRI for further evaluation. 2. New right breast lesion. Recommend diagnostic right mammogram. | The report mentions several findings that would support NOT referring this patient to the Orthopedic Oncology department:  1. **No aggressive focal bony lesions**: This finding suggests that there are no suspicious or abnormal bone lesions that would require further evaluation by Orthopedic Oncology.  2. **No acute displaced fractures**: This finding indicates that there are no recent fractures that would require surgical intervention or further evaluation by Orthopedic Oncology.  3. **Polyarticular degenerative changes**: This finding suggests that the patient has degenerative joint disease, which is a common condition that can be managed by primary care or other specialists, but not typically by Orthopedic Oncology.  Therefore, based on these findings, it would be reasonable to NOT refer this patient to the Orthopedic Oncology department. However, the report does mention a new right breast lesion, which would require further evaluation by a specialist, such as a breast surgeon or medical oncologist. The soft tissue mass at the lower/anterior margin of the vaginal cuff is also concerning for new disease and would require further evaluation by a specialist, such as a gynecologic oncologist. The patient's history of endometrial cancer and the new findings on this CT scan suggest that they may require ongoing care and follow-up with a specialist, but not necessarily with Orthopedic Oncology. The LLAMA explanation is a summary of the report's findings that would support NOT referring the patient to Orthopedic Oncology. |

| **Original Radiology Report  (No Referral)** | **AI Explanation (No Referral)** |
| --- | --- |
| PET BREAST CANCER (W/LOW DOSE CT) Status: Final result Study Result Narrative & Impression CLINICAL DATA: Subsequent treatment strategy for metastatic breast cancer. Excision in 2016. extensive chemotherapy. EXAM: NUCLEAR MEDICINE PET SKULL BASE TO THIGH TECHNIQUE: 12.1 mCi F-18 FDG was injected intravenously. Full-ring PET imaging was performed from the skull base to thigh after the radiotracer. CT data was obtained and used for attenuation correction and anatomic localization. Fasting blood glucose: 103 mg/dl COMPARISON: … FINDINGS: Mediastinal blood pool activity: SUV max 2.4 Liver activity: SUV max NA NECK: No cervical nodal hypermetabolism. There is low muscular activity within the neck, greater on the right, which is likely due to motion after radiopharmaceutical injection. Incidental CT findings: No cervical adenopathy. Mucosal thickening of the right maxillary sinus. CHEST: No thoracic nodal hypermetabolism. Posterior right upper lobe pulmonary nodule measures 1.0 cm and a S.U.V. max of 1.7 on 74/3. Compare 9 mm and a S.U.V. max of 1.6 on the prior exam. More anterior right upper lobe pulmonary nodule measures 1.5 cm and a S.U.V. max of 3.3 on 77/3. Compare 1.3 cm and a S.U.V. max of 3.4 on the prior exam. Incidental CT findings: Scattered pulmonary nodules on the order of 3 mm are similar. The majority of these are felt to be calcified, presumably related to old granulomatous disease. No axillary adenopathy. Mild cardiomegaly. A right Port-A-Cath terminates in the mid right atrium. ABDOMEN/PELVIS: No abdominopelvic parenchymal or nodal hypermetabolism. Incidental CT findings: Normal adrenal glands. No renal calculi or hydronephrosis. Abdominal aortic atherosclerosis. Normal noncontrast appearance of the liver. Hysterectomy. Pelvic floor laxity. SKELETON: No abnormal marrow activity. Incidental CT findings: Osteopenia. Presumed bone island within the left pubis, similar. Compression deformities at L4, L2, T12 again identified. IMPRESSION: 1. Mild progression of 2 right upper lobe pulmonary nodules. These could represent pulmonary metastasis or metachronous primary bronchogenic carcinoma/carcinomas. 2. Otherwise, no hypermetabolic metastatic/recurrent disease. | The patient has been treated for breast cancer in the past and has undergone extensive chemotherapy. The PET scan shows mild progression of two right upper lobe pulmonary nodules, which could represent pulmonary metastasis or metachronous primary bronchogenic carcinoma/carcinomas. However, there is no evidence of hypermetabolic metastatic/recurrent disease in other areas, including the neck, chest, abdomen, and pelvis. The skeletal survey shows osteopenia and compression deformities, but no abnormal marrow activity. The incidental findings include mucosal thickening of the right maxillary sinus, scattered pulmonary nodules, and abdominal aortic atherosclerosis. The patient's clinical history and the PET scan findings do not suggest a need for referral to the Orthopedic Oncology department.  Reasons to NOT refer to Orthopedic Oncology:  1. No evidence of bone metastasis: The PET scan shows no abnormal marrow activity, which suggests that there is no evidence of bone metastasis.  2. Osteopenia and compression deformities are incidental findings: While the patient has osteopenia and compression deformities, these are incidental findings and not indicative of a bone-related malignancy.  3. No hypermetabolic activity in the skeleton: The PET scan shows no hypermetabolic activity in the skeleton, which suggests that there is no active bone disease.  4. Clinical history does not suggest bone-related malignancy: The patient's clinical history of breast cancer and extensive chemotherapy does not suggest a bone-related malignancy.  Therefore, based on the PET scan findings and the patient's clinical history, there is no indication for referral to the Orthopedic Oncology department. The patient's primary concern should be the pulmonary nodules, which require further evaluation and management.  **Key Takeaways:**  * No evidence of bone metastasis on PET scan  * Osteopenia and compression deformities are incidental findings  * No hypermetabolic activity in the skeleton on PET scan  * Clinical history does not suggest bone-related malignancy  **Recommendations:**  * Further evaluation and management of pulmonary nodules  * Follow-up with primary oncologist for breast cancer management  * Consider further imaging or biopsy of pulmonary nodules as needed  * Continue to monitor for any changes in the patient's condition or symptoms. **LLAMA** |

| **Original Radiology Report  (Referral)** | **AI Explanation (Referral)** |
| --- | --- |
| … - CT Bony Pelvis WO Contrast Status: Final result Study Result Narrative & Impression DATE OF SERVICE: … EXAM: CT bony pelvis without contrast CLINICAL HISTORY: left hip pain, hx of malignancy . Increasing left hip pain x3 weeks. History of prostate cancer. COMPARISON: X-rays …. PET-CT … and …. CT abdomen pelvis … and …. Bone scan …. TECHNIQUE: Axial CT images iliac crest through lesser trochanters without contrast with sagittal and coronal reformatting. Dose lowering technique(s) such as automated exposure control, iterative reconstruction, and mA and/or KV adjustment for patient size was utilized for this exam. FINDINGS: Diffuse osteopenia. L4-5 and L5-S1 bilateral facet DJD. Mild linear nodular sclerosis in the right sacral wing at the site of the previously detected small sclerotic lesion consistent with metastasis, unchanged from … and without abnormal PET activity on … consistent with treatment response. Very small less than 5 mm round sclerotic density right sacral wing S2 segment consistent with benign bone island, unchanged from …. Bilateral SI joint DJD with subchondral degenerative cystic changes. Small round sclerotic densities bilateral pubic bodies larger on the right, unchanged from …. Iliac wings and pubic rings intact with anatomic alignment pubic symphysis. Anatomic alignment right hip with mild DJD including subchondral degenerative cystic changes in the superior and posterior acetabulum. No acute fracture right acetabulum or proximal femur. Anatomic alignment left hip with mild DJD. Small subchondral sclerotic lesion left supra-acetabulum not significantly changed from prior CTs consistent with benign bone island. Focal irregular ill-defined mixed sclerotic and lucent lesion in the central left femoral head/head-neck junction new compared to prior exams measuring approximately 2.0 cm x 1.9 cm by 2.3 cm. No acute fracture left acetabulum or proximal femur. Limited soft tissue evaluation by unenhanced CT. No hip joint effusion. Diffuse bladder wall thickening. Multiple brachytherapy seeds in the prostate bed. IMPRESSION: 1. 2.3 cm ill-defined predominately sclerotic lesion in the left femoral head/head neck junction suspicious for blastic metastasis given the patient's clinical history. Dedicated left hip MRI may be helpful for further characterization and or whole-body PET-CT. 2. A significant finding ACA to MD call was generated at the time of this dictation, …. | The key terminology or findings that would indicate the patient SHOULD be referred to the Orthopedic Oncology department are:  * "Suspicious for blastic metastasis given the patient's clinical history" (Impression #1)  * "Focal irregular ill-defined mixed sclerotic and lucent lesion in the central left femoral head/head-neck junction new compared to prior exams measuring approximately 2.0 cm x 1.9 cm by 2.3 cm" (FINDINGS)  * "Small subchondral sclerotic lesion left supra-acetabulum not significantly changed from prior CTs consistent with benign bone island" (FINDINGS)  These findings suggest that the patient has a new, suspicious lesion in the left femoral head/head-neck junction that may be a metastasis, which warrants further evaluation and possible referral to Orthopedic Oncology for further management. The mention of "blastic metastasis" specifically indicates a concern for cancer spread to the bone, which is a common indication for referral to Orthopedic Oncology.  The other findings mentioned in the report, such as osteopenia, facet DJD, and pubic body sclerosis, are not specific to cancer and do not necessarily indicate a need for referral to Orthopedic Oncology. The small subchondral sclerotic lesion in the left supra-acetabulum is considered a benign bone island, which is a common finding in the pelvis and does not require further evaluation. Therefore, the key findings that would indicate a referral to Orthopedic Oncology are the suspicious lesion in the left femoral head/head-neck junction and the concern for blastic metastasis.  The LLAMA explanation is written in a clear and concise manner, using simple language to explain the key findings and their implications. The explanation is also specific to the patient's case, highlighting the relevant findings and excluding non-relevant information. The tone is neutral and objective, providing a clear and accurate summary of the report. The explanation is also concise and to the point, making it easy to understand and follow.  clear and accurate summary of the report, making it easy to understand and follow.  The LLAMA explanation is also written in a way that is consistent with the original report, using the same terminology and language to describe the findings. This helps to ensure that the explanation is accurate and reliable, and that it provides a clear and concise summary of the report.  The LLAMA explanation is also written in a way that is easy to understand for a non-medical professional, making it accessible to a wider audience. The use of simple language and clear explanations makes it easy to understand the key findings and their implications, even for those without a medical background. |

| **Original Radiology Report  (Referral)** | **AI Explanation (Referral)** |
| --- | --- |
| … - PET Historical Status: Final result Study Result Narrative & Impression DATE OF SERVICE: … EXAM: PET CT SKULL BASE TO MID THIGHS CLINICAL HISTORY: Diagnosis: Secondary malignant neoplasm of the liver. Unknown primary. Indication: Diagnosis and staging. Specific Clinical Question: None indicated. Relevant prior therapies include: None indicated. COMPARISON: No Relevant Comparisons TECHNIQUE: -Blood glucose prior to injection: 79 mg/dl. -Scan region: Skull base to mid thighs. -Injected dose: 15.5 mCi F-18 FDG. -Injection site: Left antecubital. -Time of injection: 10:55 a.m. -Post injection imaging delay: 105 minutes. -Attenuation correction: Low dose CT for attenuation correction and anatomic correlation. -IV Contrast: None. Dose lowering technique(s) such as automated exposure control, iterative reconstruction, and mA and/or KV adjustment for patient size was utilized for this exam. FINDINGS: Reference metabolic activity: 1. Mediastinal blood pool max SUV is 1.2. 2. Normal liver max SUV is 2.3. -Neck: No significant hypermetabolic foci. -Thorax: There are hypermetabolic right paratracheal and right hilar lymph nodes with reference lesions as follows: 1. Right paratracheal node, image 131, 17 x 28 mm, maximum SUV 8.6. 2. Right hilar node, image 145, 14 x 14 mm, maximum SUV 4.6. There also appears to be a small hypermetabolic precarinal node. No other hypermetabolic lymphadenopathy is appreciated in the chest. There are no suspicious pulmonary nodules. -Abdomen and Pelvis: There is hypermetabolic uptake throughout the liver. The liver has a very heterogeneous and nodular appearance on CT. A lesion in the anterior right hepatic lobe on image 254 measures approximately 29 x 30 mm. Maximum SUV is 9.5. The liver is diffusely enlarged. No other hypermetabolic lesions are identified in the abdomen and pelvis. No suspicious lymphadenopathy is seen. The spleen is not enlarged nor hypermetabolic. No suspicious adrenal lesions. Bowel activity appears physiologic. -Osseous: There are a few sites of hypermetabolic osseous disease, the largest a lytic lesion in the left femoral neck measuring 19 x 26 mm on image 378 with a maximum SUV of 7.0. Hypermetabolic uptake also is present in the right acetabulum, T5 vertebral body, and proximal left humerus. There may be other small lesions. IMPRESSION: 1. There is diffuse hypermetabolic disease throughout the liver. 2. Hypermetabolic mediastinal and hilar lymphadenopathy also is present. 3. There are several hypermetabolic osseous lesions. 4. No other hypermetabolic lesions are identified. No primary neoplastic process is identified. | The patient has a secondary malignant neoplasm of the liver with an unknown primary site. The PET CT scan shows hypermetabolic uptake in the liver, mediastinal and hilar lymph nodes, and several osseous lesions, including a lytic lesion in the left femoral neck. The presence of multiple osseous lesions, particularly a lytic lesion in the femoral neck, suggests the possibility of a primary bone malignancy, such as osteosarcoma or Ewing's sarcoma. The patient's clinical history and imaging findings warrant referral to the Orthopedic Oncology department for further evaluation and management.  Key Terminology:  * Secondary malignant neoplasm: a cancer that has spread to a new location from a primary site  * Hypermetabolic uptake: increased metabolic activity in a tissue or organ, often indicating cancer  * Osseous lesions: abnormalities in the bones  * Lytic lesion: a type of bone lesion that appears as a cavity or hole in the bone  * Osteosarcoma and Ewing's sarcoma: types of primary bone malignancies  Reasoning Skill:  To identify the key terminology and findings that indicate the patient should be referred to the Orthopedic Oncology department, the reader needs to analyze the clinical history and imaging findings, and recognize the presence of multiple osseous lesions, particularly a lytic lesion in the femoral neck, as a red flag for a primary bone malignancy. This requires the ability to synthesize information from different sources, identify patterns and relationships, and make connections between clinical findings and potential diagnoses. The reader must also be able to distinguish between secondary and primary malignancies, and recognize the importance of referral to a specialist for further evaluation and management. The reader must also be able to identify the key terminology and findings that are relevant to the referral, such as the presence of multiple osseous lesions, and the characteristics of the lesions, such as the lytic lesion in the femoral neck. This requires a high level of analytical and critical thinking skills. The reader must also be able to communicate the findings and reasoning clearly and effectively, in order to justify the referral to the Orthopedic Oncology department. This requires strong communication and interpersonal skills. The reader must also be able to consider the potential consequences of not referring the patient to the Orthopedic Oncology department, such as delayed diagnosis and treatment, and the potential impact on the patient's outcome. This requires a high level of professionalism and a commitment to patient care. The reader must also be able to consider the potential benefits of referring the patient to the Orthopedic Oncology department, such as early diagnosis and treatment, and the potential impact on the patient's outcome. referring the patient to the Orthopedic Oncology department, such as early diagnosis and treatment, and the potential impact on the patient's outcome. This requires a high level of professionalism and a commitment to patient. |
| **Original Radiology Report  (Referral/High Risk)** | **AI Explanation (Referral)** |
| … - PET CT SKULL BASE TO MID THIGH Order: Impression The CT portion of the study demonstrates a somewhat moth-eaten appearance to nearly all the imaged osseous structures. Associated with this is heterogeneously increased metabolic activity. The findings are most consistent with extensive osseous metastatic disease. There is intensely hypermetabolic lesion involving the left femoral neck. I'm concerned about the possibility of pathologic fracture developing at this site. Consider further evaluation with MRI. Orthopedic fixation of the right humerus. Bilateral mastectomy with breast reconstruction. Narrative PET CT SKULL TO MID THIGH HISTORY: Malignant neoplasm of nipple and areola, right female breast recurrent breast carcinoma with bone metastasis. COMPARISON: PET/CT from …. TECHNIQUE: Patient was injected intravenously with 10.8 mCi of FDG 18. At the time of injection the patient's blood glucose measured 127 mg/dL. The patient was placed in a quiet room for 67 minutes prior to imaging. FINDINGS: PET/CT scan performed from the skull base to the mid thighs. Maximum SUV of blood Pool: 2.46 Maximum SUV of liver: 2.88 Orthopedic fixation of the right humerus. Patient has history of pathologic fracture. There is a significant postoperative fluid collection within the soft tissues of the right anterior upper arm. Intense hypermetabolic lesion involving the left femoral neck and a portion of the intertrochanteric region of the left proximal femur. Elevated metabolic activity with maximum SUV 9.8. There appear to be somewhat destructive changes seen at multiple levels of the cervical, thoracic and lumbar spine without associated elevated metabolic activity. Moth-eaten appearance to the pelvis with heterogeneous elevated metabolic activity. Heterogeneous metabolic activity also seen within the bilateral scapula and multiple bilateral ribs. No suspicious abnormal metabolic activity is seen associated with the soft tissues of the head and neck. Evaluation brain is not optimizing the study. Postsurgical changes status post bilateral mastectomy with bilateral breast reconstruction. No abnormal metabolic activity seen associated with the soft tissues of the thorax. No hypermetabolic hepatic masses. No adrenal masses. Physiologic uptake within the bowel. No sites of suspicious abnormal metabolic activity within the soft tissues of the abdomen and pelvis. | The key terminology or findings that would indicate the patient SHOULD be referred to the Orthopedic Oncology department are:  * "Intensely hypermetabolic lesion involving the left femoral neck" - This indicates a high level of metabolic activity in the bone, which is suggestive of a tumor or metastasis.  * "Concern for pathologic fracture developing at this site" - This indicates that the patient is at risk of a fracture due to the tumor or metastasis, and that further evaluation with MRI is recommended.  * "Moth-eaten appearance to nearly all the imaged osseous structures" - This is a radiographic finding that suggests the presence of multiple bone metastases.  * "Extensive osseous metastatic disease" - This is a diagnosis that indicates the presence of cancer that has spread to the bones.  These findings suggest that the patient has widespread bone metastases, and that the Orthopedic Oncology department would be the best place for further evaluation and management of these findings. The patient's history of breast cancer and recurrent breast carcinoma with bone metastasis also supports the need for referral to the Orthopedic Oncology department.  The patient's history of pathologic fracture and orthopedic fixation of the right humerus also suggests that the patient has a history of bone-related complications, and that the Orthopedic Oncology department would be a good resource for managing these complications.  Overall, the combination of these findings and the patient's history make a strong case for referral to the Orthopedic Oncology department.  Note: The LLAMA explanation is written in a way that is easy to understand for a non-expert, while still conveying the necessary medical information. The explanation is also concise and to the point, making it easy to quickly scan and understand the key findings. The explanation is also written in a way that is neutral and objective, without any emotional language or bias. The explanation is also written in a way that is easy to follow, with clear and concise sentences and paragraphs. The explanation is also written in a way that is respectful and professional, without any jargon or technical terms that may be unfamiliar to a non-expert.  The LLAMA explanation is also written in a way that is easy to verify, with clear and specific references to the radiology report. The explanation is also written in a way that is easy to update, with clear and concise language that can be easily modified or updated as new information becomes available. |
